# Transcriptome-Wide Analysis of N6-Methyladenosine Modification in the Liver of Geese at Different Growth Stages

**DOI:** 10.3390/ani16060981

**Published:** 2026-03-20

**Authors:** Chuan Li, Jintao Wu, Shuibing Liu, Wentao Zhang, Jing Liu, Sanfeng Liu, Biao Chen

**Affiliations:** 1 College of Life Sciences, Jiangxi Science and Technology Normal University, Nanchang 330013, China; 1020241213@jxstnu.edu.cn; 2 College of Animal Science and Technology, Jiangxi Agricultural University, Nanchang 330045, China; wjt884007846@stu.jxau.edu.cn (J.W.); 20241024012@stu.scau.edu.cn (S.L.); zwt0821@163.com (W.Z.); jingliuexon@163.com (J.L.); lsf3318@jxau.edu.cn (S.L.); 3 Poultry Institute, Jiangxi Agricultural University, Nanchang 330045, China

**Keywords:** m^6^A modification, molecular mechanisms, liver, growth stages, geese

## Abstract

N6-methyladenosine (m^6^A) is a reversible RNA modification that dynamically regulates gene expression by modulating RNA stability, splicing, nuclear export, translation, and maturation—thereby orchestrating organismal development. In birds, including geese, the liver is a multi-functional organ central to metabolic regulation. Studies on the dynamic patterns of RNA m^6^A modifications during healthy liver growth and development remain limited. Here, we performed integrative methylated RNA immunoprecipitation sequencing (MeRIP-seq) and RNA sequencing (RNA-seq) on liver tissues from geese at three biologically defined stages: post-hatch day 0 (0 week, P), fast growth (10 weeks, F), and sexual maturation (30 weeks, S). The level of m^6^A modification in total RNA extracted from liver tissues was higher in P than in F samples. Compared with other groups, the S group recorded the lowest m^6^A modification. In addition, 1641, 668, and 558 m^6^A peaks were differentially modified in the P, F, and S groups, respectively. The m^6^A peaks in the liver of the three groups were mainly enriched in the coding sequence and 3′ untranslated region. Moreover, integrated multi-omics analysis (MeRIP-seq and RNA-seq), combined with protein–protein interaction networks analysis, identified *CDK1* as a core cell cycle regulator and *IGF2BP3*—a well-established m^6^A reader—as a consistently differentially expressed gene across all developmental stages. The m^6^A-regulated cell cycle, p53 signaling pathway, and pyrimidine metabolism pathway were identified in liver tissue as novel potential targets for controlling geese growth and metabolism. Together, these findings shed light on the dynamic regulation of RNA methylation during distinct growth phases in geese and advance our understanding of epigenetic mechanisms underlying poultry liver development.

## Simple Summary

This study addresses the limited understanding of dynamic RNA m^6^A methylation during healthy liver development in birds, using geese as a model. Our objective was to profile transcriptome-wide m^6^A modifications across key postnatal stages (newly hatched, fast growth, and sexual maturation) via MeRIP-seq and RNA-seq. Results revealed stage-specific m^6^A levels and differentially methylated peaks, predominantly in coding sequences and 3′UTRs. Integrated bioinformatic analysis identified cyclin-dependent kinase 1 (CDK1) as a potential central regulator of the cell cycle and insulin-like growth factor 2 mRNA-binding protein 3 (IGF2BP3) as a key m^6^A reader, thereby linking m^6^A-mediated RNA modifications to critical biological processes—including cell cycle progression, p53 signaling activation, and pyrimidine metabolism—during liver development. These findings elucidate epigenetic mechanisms governing poultry liver growth and metabolism, providing potential molecular targets for improving poultry health and productivity, thereby offering value to avian research and agricultural practices.

## 1. Introduction

Avian development is a complex and highly regulated process, encompassing rapid growth and organ development during early life stages, followed by maturation and maintenance as birds reach adult age [1]. Dynamic molecular mechanisms that orchestrate gene expression and cellular processes underpin this intricate developmental trajectory. Among these mechanisms, RNA chemical modifications have emerged as crucial regulators of gene expression, influencing RNA stability, maturation, splicing, nuclear export, and translation, ultimately affecting organismal development [2,3].

N6-methyladenosine (m^6^A) is one such RNA modification that has garnered increasing attention in recent years [2,4,5,6,7]. It involves methylation of the adenosine residue within RNA molecules and plays a pivotal role in post-transcriptional regulation [3]. The m^6^A modification is installed by the writer complex, comprising METTL3, METTL14, WTAP, RBM15, VIRMA, and ZC3H13, and removed by the eraser enzymes FTO and ALKBH5 through demethylation [2]. The roles and functions of m^6^A are mediated by reader proteins (m^6^A-binding proteins), which play pivotal roles in regulating RNA metabolism, including YTHDC, YTHDF, and IGF2BP3 [8,9]. Among poultry, m^6^A modification has been broadly studied. It is widely acknowledged to influence various biological developmental processes, including ovarian development [10], differentiation and development of reproductive glands [11], muscle development [12,13], and the proliferation and differentiation of preadipocytes [14]. However, our understanding of m^6^A modification in avian species, particularly its dynamics across different developmental stages of the liver, remains limited.

The liver in birds, including geese, is a multi-functional organ. It plays an essential role in glucose metabolism, energy homeostasis, lipid metabolism, nutrient storage, amino acid metabolism, detoxification, vitamin metabolism, and the support of diverse metabolic processes essential for adaptation and survival under varying environmental conditions [15,16]. These processes are regulated by different factors, such as genetics, nutrition, and environment [16,17]. Geese (*Anser domesticus*) serve as a highly valuable avian model for investigating the dynamics of m^6^A modifications during liver development. In humans and most mammals, excessive hepatic fat accumulation typically leads to liver steatosis and the development of non-alcoholic fatty liver disease. In contrast, geese—domesticated waterfowl derived from migratory birds—possess distinctive hepatic lipid metabolism characteristics that confer strong tolerance, resistance, and regenerative capacity against steatosis, and even pronounced fat deposition in the goose liver does not result in pathological lesions and can spontaneously revert to a histologically normal state [18]. Previous studies have demonstrated that m^6^A modification is involved in the regulation of goose muscle development [19,20], yet the regulatory mechanism underlying its role in liver development remains unclear. As one of China’s important local breeds, the Sichuan white goose [21], together with its counterpart, the Tianfu meat goose, is widely known as a valuable source ingredient for foie gras production. These birds exhibit marked growth and physiological changes during their first post-hatch stages, culminating in complete maturation as they reach adulthood. Although the molecular processes controlling these developmental shifts are undoubtedly intricate, the role of m^6^A modification in these conditions remains largely unexplored.

We hypothesized that there are significant differences in the transcriptome and epitranscriptome of goose livers across different growth stages, and that these differences can be revealed by MeRIP-seq and RNA-seq analyses. Previous studies have identified the 10-week-old post-hatching stage as a critical developmental period in male geese, including the Sichuan white goose [22]. In contrast, approximately 30 weeks of age marks the onset of sexual maturity [22]. He et al. investigated the dynamic patterns of m^6^A methylation in porcine liver across three developmental stages: the neonatal period (0 days), the lactation period (21 days), and the adult period (2 years) [23]. Similarly, by focusing on 0-week-old (post-hatch day 0), 10-week-old (fast-growth stage), and 30-week-old (sexual-maturation stage) geese, we aim to elucidate how m^6^A modifications vary across developmental stages of the liver transcriptome. Our investigation examines the prevalence and distribution of RNA m^6^A modifications across growth stages and explores potential links to the goose liver developmental stages. These findings may offer valuable insights into the epitranscriptomic regulation of hepatic maturation and function in avian species.

## 2. Materials and Methods

### 2.1. Ethics Approval and Consent to Participate

All animals used in this experiment conformed with the ethical principles of the Jiangxi Agricultural University’s ethical norms and regulations (JXAULL-2017002), and followed the ethical guidelines for animal experimentation established by Jiangxi Science and Technology Normal University. All methods are reported in accordance with ARRIVE (https://arriveguidelines.org) guidelines.

### 2.2. Experimental Animal and Tissue Collection

Male Sichuan white geese (*Anser domesticus*) that were hatched from the same batch of fertilized eggs acquired from the Sichuan Agricultural University’s Waterfowl Breeding Experimental Farm (Ya’an, China) were used in this project. The birds were reared under standardized environmental and nutritional conditions, exhibiting normal growth and development [22]. The livers of nine geese were sampled (three samples at each stage) at 0 weeks old (post-hatch day 0, P group), 10 weeks old (fast-growth stage, F group), and 30 weeks old (sexual-maturation stage, S group). Liver tissues were collected and snap-frozen in liquid nitrogen before being kept at −80 °C.

### 2.3. RNA Extraction for MeRIP-Seq and RNA-Seq

Total RNA was extracted and purified according to the manufacturer’s instructions using the TRIzol reagent (Invitrogen, Carlsbad, CA, USA). The NanoDrop ND-1000 was used to determine the amount and quality of RNA obtained in each sample (NanoDrop, Wilmington, DE, USA). The integrity of the RNA was determined using a Bioanalyzer 2100 (Agilent, Santa Clara, CA, USA) and confirmed by electrophoresis on a denaturing agarose gel. Poly (A) RNA was separated from 50 µg total RNA in two rounds of purification using Dynabeads Oligo (dT)25-61005 (Thermo Fisher Scientific, Waltham, MA, USA). Following that, the poly(A) RNA was fragmented for 7 min at 86 °C using a Magnesium RNA Fragmentation Module (NEB, Ipswich, MA, USA). After that, 10% of RNA fragments were saved as “input”. After 2 h of incubation at 4 °C with a m^6^A-specific antibody (Synaptic Systems, Göttingen, Germany), the fragmented RNA was harvested (50 mM Tris-HCl, 750 mM NaCl, and 0.5 percent Igepal CA-630). Along with “input”, IP RNA was collected for MeRIP-seq library construction. For RNA-seq, the input libraries from the same IP experiments were used for sequencing.

### 2.4. Library Constructions and Sequencing

After reverse transcription of the IP and input RNA with SuperScript TM II Reverse Transcriptase (Invitrogen, USA), the resulting cDNAs were used to synthesize uracil-labeled second-stranded DNAs using *E. coli* DNA polymerase I (NEB, USA), RNase H (NEB, USA), and dUTP Solution (Thermo Fisher, USA). The blunt ends of each strand were next treated with an A-base to prepare them for ligation to the indexed adapters. The fragments were ligated to adapters, and size selection was done using AMPure XP beads. Following treatment of the uracil-labeled second-stranded DNAs with the heat-labile UDG glycosylase (UDG, NEB, USA), the ligated products were amplified using PCR under the following conditions: initial denaturation at 95 °C for 3 min; 8 cycles of denaturation at 98 °C for 15 s, annealing at 60 °C for 15 s, and extension at 72 °C for 30 s; and final extension at 72 °C for 30 s. Finally, according to the vendor’s suggested procedure, we sequenced the paired-end fragment (PE150) on an Illumina Novaseq^TM^ 6000 (LC-Bio, Hangzhou, China).

### 2.5. MeRIP-Seq Data Analysis

With the default setting, the fastp program (https://github.com/OpenGene/fastp, accessed on 3 June 2023) was used to eliminate reads that included adapter contamination, low-quality bases (quality score < 20), and reads containing ambiguous bases (N). To map the clean reads to the goose (*Anser cygnoides*) reference genome, the NCBI database (https://ftp.ncbi.nlm.nih.gov/genomes/all/GCF/000/971/095/GCF_000971095.1_AnsCyg_PRJNA183603_v1.0, accessed on 7 June 2023) and HISAT2 (http://daehwankimlab.github.io/hisat2, accessed on 7 June 2023) (version: v96) were adopted. The mapped reads from both IP and input libraries were analyzed using the R package exomePeak (version 2.6.0) (https://bioconductor.org/packages/exomePeak, accessed on 15 June 2023), which detects m^6^A peaks in bed or bigwig format for display using the IGV program (http://www.igv.org). The candidate peak regions on the genome were extended to form modeling areas of a specified length. Based on the unique aligned reads within these areas, we employed a Poisson distribution model to perform statistical testing and calculate the *p*-values for the candidate peak regions. A default threshold of *p* < 0.05 was set, with regions below this threshold considered as significant peaks. MEME (http://meme-suite.org) and HOMER (http://homer.ucsd.edu/homer/motif, accessed on 23 June 2023) were used to discover new and recognized motifs, respectively, and then to annotate the motif. Using the R package ChIPseeker (version 1.34.0) (https://bioconductor.org/packages/ChIPseeker, accessed on 29 June 2023), peaks were pinpointed by their intersection with genes. Then, for transcript expression analysis, fragments per kilobase of exon model per million mapped fragments (FPKM) values for all genes were calculated using StringTie (v2.1) (https://ccb.jhu.edu/software/stringtie, accessed on 4 July 2023) with default parameters in the IP and input libraries. Significantly differential m^6^A peaks were identified using a threshold of *p* < 0.05 and fold change > 1.5.

For the input RNA, sequencing was also performed using the Illumina NovaseqTM 6000 platform in PE150 mode. Following sequencing, paired-end reads were obtained. The fastp program was used for data preprocessing, including Q30 quality control and adapter trimming, resulting in high-quality reads. HISAT2 (v2.2.1) was used to align the high-quality reads to the goose reference genome. The StringTie was then employed to obtain raw counts at the gene level for the mRNA expression profile. The edgeR was utilized to normalize the expression levels across all samples to FPKM values and to calculate fold changes and *p*-values between two groups. Differentially expressed genes (DEGs) were identified with |log2 fold change| > 1 and a *p*-value < 0.05 as thresholds, followed by GO and KEGG enrichment analysis.

### 2.6. qPCR Assay

For the RNA-seq validation, reverse transcription of total RNAs was performed using the Monad MonScript^TM^ All-in-One Kit with DNase (Biopro, Shanghai, China). The expression level was evaluated using a final volume of 10 µL of 2 × T5 Fast qPCR Mix (TsingKe, Beijing, China). Internal control was *GAPDH*. The following protocol was used for the qPCR reaction: 95 °C for 3 min; 40 cycles of 95 °C for 10 s; Tm for 30 s; and fluorescence acquisition at 65–95 °C. Each sample was analyzed in triplicate, and the 2^−∆∆CT^ tactic was used to assess the relative RNA expression level. Table S18 contains the sequences and information of the qPCR primers. Statistical analyses for quantitative qPCR data were performed using a two-tailed Student’s *t*-test, with significance thresholds set at * *p* < 0.05, ** *p* < 0.01, and *** *p* < 0.001. The qPCR data was presented as mean ± standard error of the mean (S.E.M.).

## 3. Results

### 3.1. Overview of Sequencing-Data

MeRIP-seq and RNA-seq collectively generated 116.12 gigabytes (GB) of raw sequencing data. Immunoprecipitation (IP) samples yielded an average of 35,924,972 raw reads per library, whereas input samples yielded an average of 36,518,411 raw reads per library (Table 1). Following data quality control, an average of 35,923,736 high-quality (clean) reads were retained from IP libraries and 36,517,586 from input libraries. The Q30 score—representing the proportion of bases with a base-calling error probability ≤0.001—exceeded 90% across all libraries, confirming high sequencing accuracy and suitability for downstream methylome and transcriptome analyses (Table 1).

**Figure 1:**
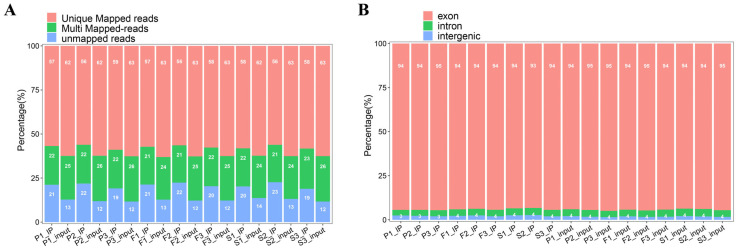
Percentage (%) of mapped reads of the MeRIP-seq sequencing data. (**A**) Comparison of read distribution in IP and input samples, categorized as Unique mapped reads, Multi-mapped reads, and Unmapped reads. (**B**) Distribution of valid reads compared to exon, intron, and intergenic regions of the reference genome.

Following data quality control, the clean reads were aligned to the reference goose genome. Alignment results revealed that, on average, 76.3% of clean reads were mapped to the reference goose genome. Of these, 54.5% were uniquely aligned to the genome, while 21.8% mapped to multiple genomic locations (Figure 1A). Analysis of read mapping positions revealed that an average of 91.27% of aligned reads were distributed in the exonic regions of genes, while 4.33% and 4.40% of reads mapped to introns and intergenic regions, respectively (Figure 1B).

### 3.2. Transcriptome-Wide Distribution of m^6^A Peaks and Motif Enrichment Analysis

**Figure 2:**
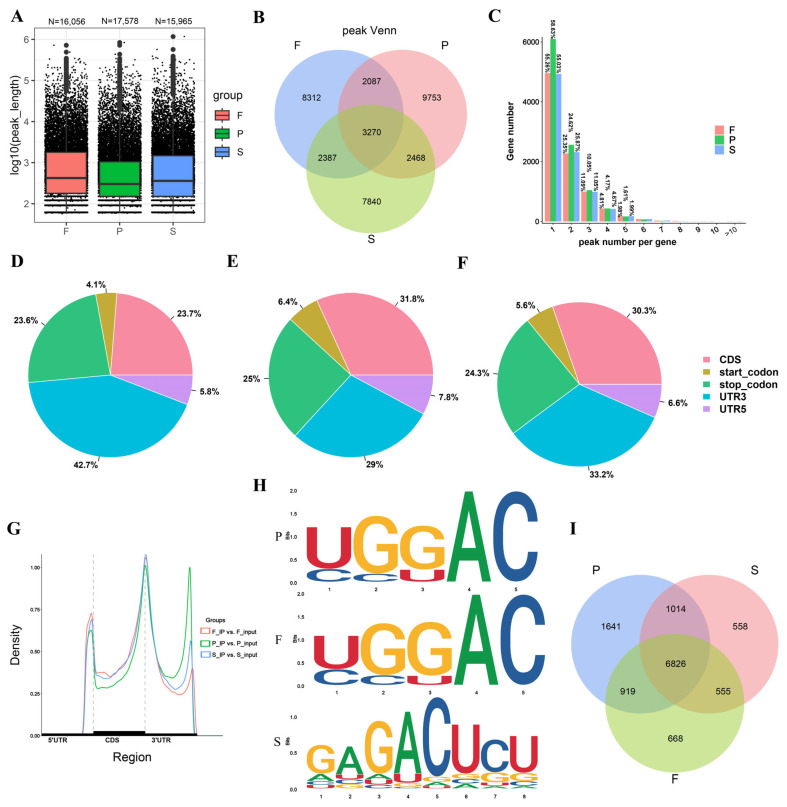
Transcriptome-wide distribution of m^6^A peaks and motif enrichment analysis. (**A**) Peak length of m^6^A in the P, F, and S groups. (**B**) Cross-comparison analysis of peaks shared in the three groups. (**C**) The percentage (%) of m^6^A peak distribution per gene. Percentage (%) of m^6^A peak distribution in CDS, start codon, stop codon, 3′UTR, and 5′UTR in groups P (**D**), F (**E**), and S (**F**). (**G**) m^6^A peak density across gene transcripts divided into 3 segments: 5′UTR, CDS, and 3′UTR. (**H**) Consensus motif of the m^6^A peak in the goose liver of groups P, F, and S. (**I**) m^6^A peak-associated-gene cross-comparison analysis of the three growth phases.

After aligning the reads to the reference genome, we performed genome-wide peak calling. In the P, F, and S groups, 17,578, 16,056, and 15,965 peaks were identified, respectively (Figure 2A). The length distribution of m^6^A peaks across the three groups was also presented in Figure 2A. Cross-comparison analysis of peaks shared in the three groups showed that 3270 peaks were detected among all three groups, whereas 9753 peaks were unique to the P group, 8312 to the F group, and 7840 to the S group (Figure 2B). Among the three groups, a significant portion (more than 55%) of m^6^A peaks were found within a single gene. Around 25% of genes exhibited two m^6^A peaks within the same gene, while approximately 11% of genes showed three m^6^A peaks (Figure 2C, Table S1). Annotations of peak positions within genes revealed that peaks were primarily concentrated at 3′UTR regions, CDS regions, stop codon, and start codon (Figure 2D–G). Notably, the 3′UTR region of the P group exhibited higher m^6^A peak enrichment compared to the F and S groups (Figure 2D). Subsequently, we conducted motif sequences and revealed that common motifs in all three groups included the RRACH motif (the R, a purine (A or G); and the H, a non-guanine base (A, C, or U). Additionally, compared to the P and F groups, the S group showed greater variability in the motif sequences (Figure 2H). Upon further analysis of cross-comparison of genes associated with the identified peaks, we found that 6826 peaks associated with genes were common to all three groups, while 1641, 668, and 558 peaks were uniquely expressed in the P, F, and S groups, respectively (Figure 2I).

### 3.3. Differential and Functional Analysis of m^6^A Peaks at Three Goose Liver Developmental Stages

**Figure 3:**
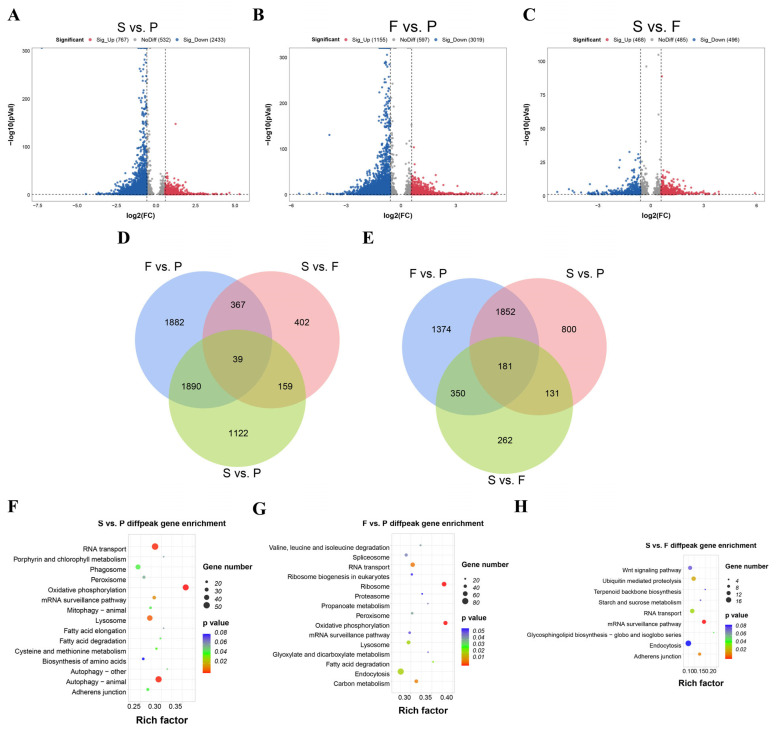
Differential m^6^A methylation profiles of goose liver at three key growth stages. Volcano plot of differential peaks (methylation level) in the S vs. P (**A**), F vs. P (**B**), and (**C**) S vs. F groups. (**D**) Venn diagrams showing differential m^6^A peaks and (**E**) differential m^6^A peak-related genes in the three cross-comparison groups. Functional enrichment analysis of differential m^6^A peak-associated genes in S vs. P (**F**) top 15 KEGG pathways, F vs. P (**G**) top 15 KEGG pathways, and S vs. F (**H**) pathways with *p* < 0.1 is presented.

The differential methylation status analysis demonstrated that among the three comparisons, S vs. P, F vs. P, and S vs. F, 767, 1155, and 468 significantly upregulated peaks and 2433, 3019, and 496 significantly downregulated peaks were identified, respectively (Figure 3A–C). In cross-comparison analysis, 1882, 402, and 1122 m^6^A peaks were uniquely found to be in the groups F vs. P, S vs. F, and S vs. P, respectively (Figure 3D). However, all three groups shared 39 unique m^6^A peaks. In addition, peak-associated genes of the cross-comparison groups—1374, 262, and 800 m^6^A peak genes—were uniquely found to be in the groups F vs. P, S vs. F, and S vs. P, respectively (Figure 3E), whereas all three groups shared 181 unique m^6^A peak genes. Based on KEGG pathway enrichment analysis, comparing between S vs. P, differentially methylated genes (DMGs) were mostly enriched to RNA transport, nutrient metabolism (porphyrin and chlorophyll metabolism), and fat metabolism pathways (Figure 3F, Table S2). Comparing between F vs. P, DMGs were mostly enriched in oxidative phosphorylation, valine leucine and isoleucine degradation, and fatty acid degradation (Figure 3G, Table S3). Comparing between S vs. F, DMGs were mostly enriched to the Wnt signaling pathway, ubiquitin-mediated proteolysis, mRNA surveillance pathway, etc. (Figure 3H, Table S4). It is important to mention that all cross-comparison groups shared top enrichment of the RNA transport and mRNA surveillance pathways.

### 3.4. Identification and Functional Analysis of DEGs

**Figure 4:**
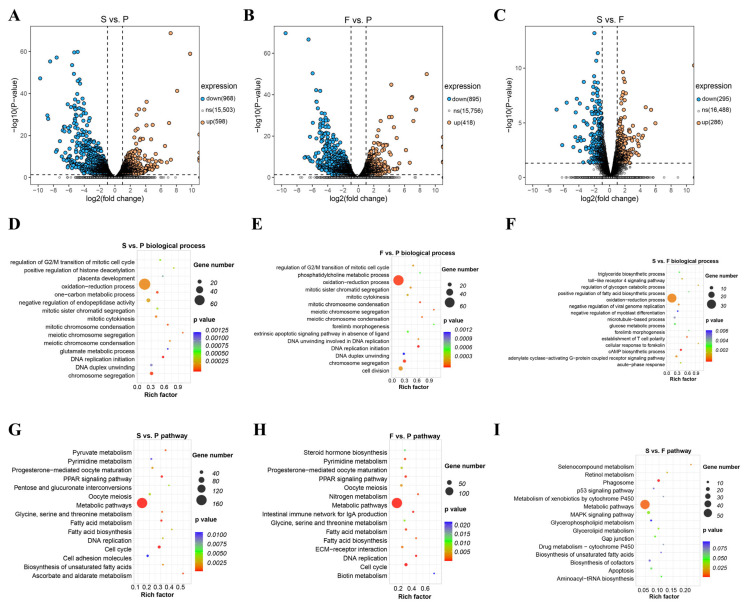
Identification and functional analysis of DEGs at three growth stages. Volcano plot of DEGs in the (**A**) S vs. P, (**B**) F vs. P, and (**C**) S vs. F groups. GO biological process results in (**D**) compared S vs. P, in (**E**) F vs. P, and in (**F**) S vs. F groups. The top 15 GO biological processes are shown. KEGG pathway results of DEGs in (**G**) S vs. P, in (**H**) F vs. P, and in (**I**) S vs. F comparison groups. The top 15 KEGG pathways are shown.

For the RNA-seq data of the input libraries in the MeRIP experiments at three time points, we employed a standard RNA-seq data analysis workflow for the selection and enrichment analysis of differentially expressed genes (DEGs). Following FPKM value calculation and differential gene analysis, we identified a total of 968 downregulated genes and 598 upregulated genes in the S vs. P comparison group (Figure 4A). In the F vs. P group, we identified 895 downregulated genes and 418 upregulated genes (Figure 4B). As for the S vs. F comparison group, a total of 563 DEGs were identified, including 295 downregulated and 268 upregulated genes (Figure 4C).

Using GO enrichment analysis, we found that in the S vs. P and F vs. P groups, DEGs were significantly enriched in biological processes related to cell cycle and cell proliferation, such as regulation of G2/M transition of mitotic cell cycle, mitotic sister chromatid segregation, DNA replication initiation, chromosome segregation, and mitotic chromosome condensation (Figure 4D,E; Tables S5 and S6), while the S vs. F group showed that GO enrichment was high in the triglyceride biosynthetic process. All cross-comparison groups shared higher GO biological process enrichment in the oxidation reduction process (Figure 4F; Table S7).

KEGG enrichment analysis revealed that in the S vs. P and F vs. P comparison groups, DEGs were significantly enriched in pathways related to lipid metabolism (PPAR signaling pathway, fatty acid metabolism, fatty acid biosynthesis, steroid hormone biosynthesis) and cell-proliferation-related pathways (cell cycle, DNA replication) (Figure 4G,H; Tables S8 and S9). In contrast, the S vs. F comparison group was mainly enriched in metabolism-related pathways such as selenocompound metabolism, biosynthesis of unsaturated fatty acids, cell-apoptosis-related pathway (p53 signaling pathway), retinol metabolism, and immune-related pathway (phagosome) (Figure 4I; Table S10).

The RNA-seq results of the goose liver at three developmental stages indicate that the liver in the P group is primarily involved in the cell proliferation phase. In contrast, the livers in the F and S groups are mainly involved in functions related to lipid metabolism, glucose metabolism, and immune-related pathways.

### 3.5. Integration Analysis of MeRIP-Seq and RNA-Seq Data

**Figure 5:**
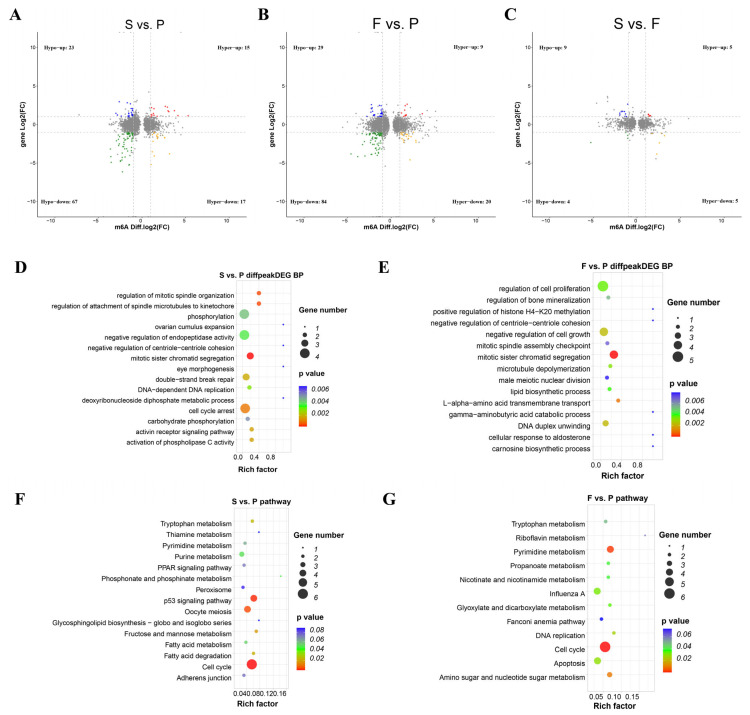
Association analysis of DMGs and DEGs in liver samples of the S vs. P, F vs. P, and S vs. F groups. Gene distributions with significant alterations in both gene expression and m^6^A-modified levels in the (**A**) S vs. P, (**B**) F vs. P, and (**C**) S vs. F groups. Hyper-up: m^6^A peak and mRNA level upregulation; Hyper-down: m^6^A peak upregulation and mRNA level downregulation; Hypo-up: m^6^A peak downregulation and mRNA level upregulation; Hypo-down: m^6^A peak and mRNA level downregulation. GO biological process results of genes with significant alterations in both gene expression and m^6^A-modified levels in (**D**) compared S vs. P and in (**E**) F vs. P. Top 15 GO biological processes are shown. KEGG pathway enrichment of both gene expression and m^6^A-modified levels in the (**F**) S vs. P (top 15 KEGG pathways are shown) and (**G**) F vs. P groups (pathways with *p* < 0.1 are shown).

We next analyzed the expression/modification correlation between the MeRIP-seq and RNA-seq data. In comparisons of S vs. P, F vs. P, and S vs. F, we identified a total of 32 (hyper-up: 15, hyper-down: 17), 29 (hyper-up: 9, hyper-down: 20), and 10 (hyper-up: 5, hyper-down: 5) hyper-methylated peaks, respectively. Also, in total, 90 (hypo-up: 23, hypo-down: 67), 113 (hypo-up: 29, hypo-down: 84), and 13 (hypo-up: 9, hypo-down: 4) hypo-methylated peaks were found for groups S vs. P, F vs. P, and S vs. F, respectively (Figure 5A–C; Tables S11–S13). Using GO biological process analysis, the comparison of the S vs. P group determined that m^6^A peaks associated with DEGs were mostly involved in mitotic sister chromatid segregation, regulation of mitotic spindle organization, regulation of attachment of spindle microtubules to kinetochore, and cell cycle arrest, whereas F vs. P was mostly involved in mitotic sister chromatid segregation, L-alpha-amino acid transmembrane transport, DNA duplex unwinding, negative regulation of cell growth and microtubule depolymerization (Figure 5D,E; Tables S14 and S15).

Using KEGG analysis, the comparison of the S vs. P group determined that m^6^A peaks associated with DEGs were mostly implicated in the cell cycle and p53 signaling pathway, whereas F vs. P was mostly concerned with cell cycle, pyrimidine metabolism and amino sugar and nucleotide sugar metabolism (Figure 5F,G; Tables S16 and S17).

### 3.6. Analysis of Differentially Methylated Genes (DMGs) and Differentially Expressed Genes (DEGs)

**Figure 6:**
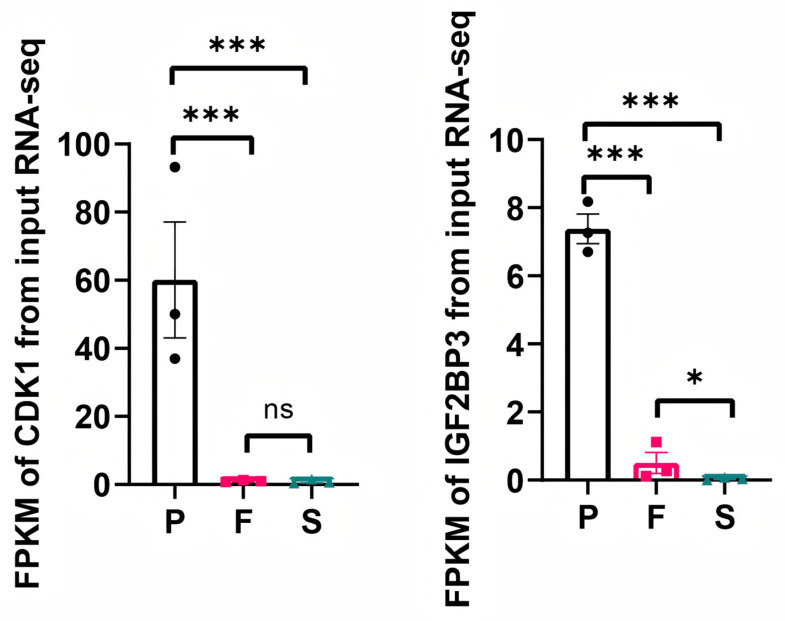
The expression levels of *CDK1* and *IGF2BP3* in the P, F and S groups. * *p* < 0.05, and *** *p* < 0.001. ns: not significant (*p* ≥ 0.05).

**Figure 7:**
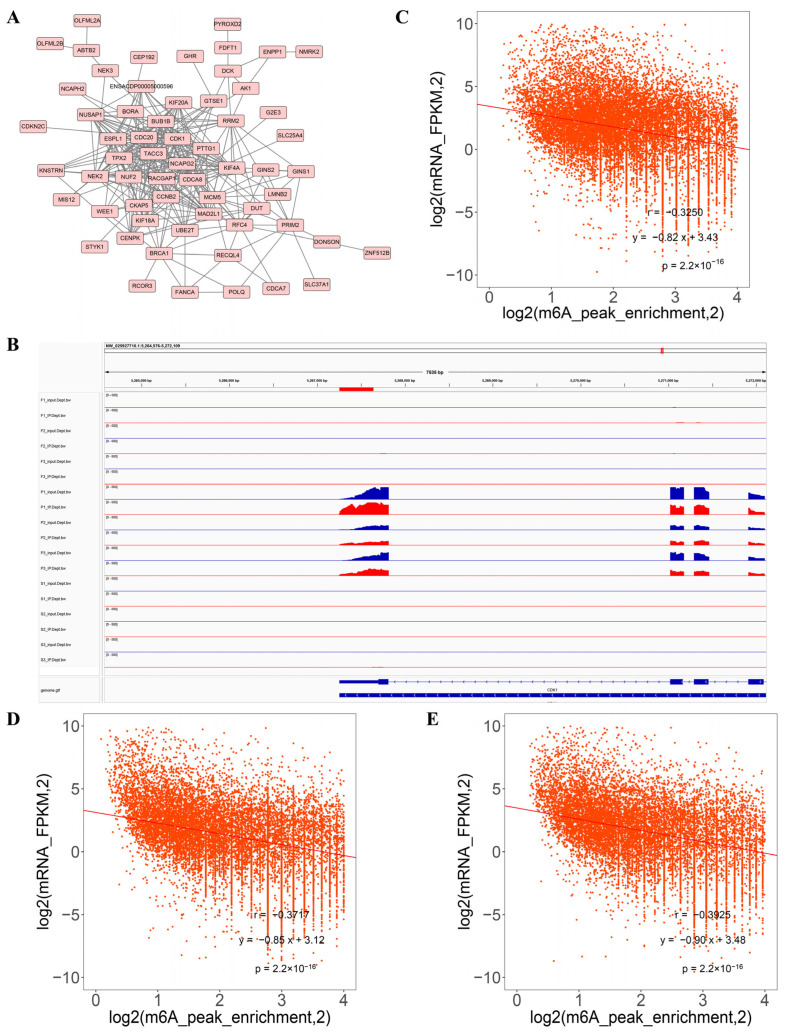
Analysis of DMGs and DEGs. (**A**) CDK1 was identified as the core protein in the protein interaction network analysis with genes that exhibited significant differences in both methylation status and expression levels. (**B**) IGV browser showing the methylation change of CDK1. Correlation analysis between m^6^A methylation peak and mRNA expression in S vs. P (**C**), F vs. P (**D**), and S vs. F (**E**) groups.

We next aimed to identify core genes at the intersection of the DMG and DEG datasets that are potentially involved in liver development across various stages. This analysis revealed that cyclin-dependent kinase 1 (*CDK1*) was strongly modified by m^6^A and differentially expressed (S vs. P and F vs. P) during the three developmental stages (Figure 6). Meanwhile, RNA-seq screening identified *IGF2BP3*, an m^6^A modification recognition protein, as a commonly differentially expressed gene during the three developmental stages in the liver (Figure 6). In addition, using the STRING (https://cn.string-db.org/) biological database, the analysis showed that *CDK1* was identified as the potential core protein in the protein–protein interaction network with the genes that showed significant alterations in both gene expression and m^6^A-modified levels in the three comparisons (Figure 7A). Integrative genomics viewer (IGV) (https://igv.org/) browser showed the methylation changes of *CDK1* during the liver development. The m^6^A modification existed in the post-hatch stage (0 weeks old), while 10 weeks old (fast-growth stage) and 30 weeks old (sexual-maturation stage) did not show any m^6^A modification (Figure 7B). Moreover, the integrated analysis showed negative correlations between m^6^A methylation peaks and gene-expression-level-associated m^6^A peaks in the three comparisons (Figure 7C–E).

### 3.7. Validation of RNA-Seq Data

**Figure 8:**
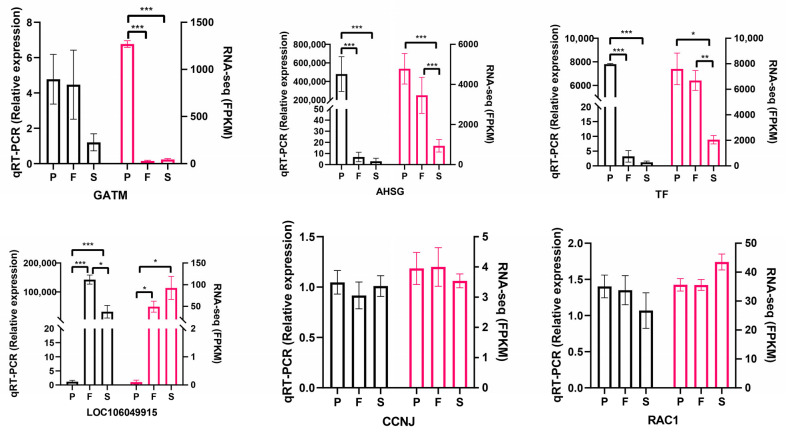
qPCR validation of some DEGs in the liver of groups P, F, and S. * *p* < 0.05, ** *p* < 0.01, and *** *p* < 0.001.

The reliability of the sequencing results was further confirmed by examining significant differences in the mRNA levels of six DEGs—representing upregulated and downregulated patterns across distinct liver developmental stages—as shown in Figure 8. This consistency in trends aligns with the patterns observed in the RNA-seq data, further strengthening the trustworthiness of our findings.

## 4. Discussion

This investigation into m^6^A modification patterns in geese livers across different growth stages has made a significant contribution to the understanding of avian developmental biology and RNA epigenetics. Three key developmental phases were studied: post-hatch day 0 (P), fast-growth stage (F), and sexual-maturation stage (S), enabling an extensive exploration of m^6^A dynamics in the geese’s development. This study is comparable to the previous research that investigated the dynamic patterns of m^6^A methylation in pig liver across three developmental stages: the neonatal period (0 days), the lactation period (21 days), and adulthood (2 years) [23]. In this study, 9753, 8312, and 7840 unique m^6^A-modified peaks were identified in the liver tissue samples of the P, F, and S groups, respectively. These differences among the groups in the numbers of m^6^A-modified peaks confirm the involvement of m^6^A in geese’s liver development.

One of the vital findings of this study was the characterization of m^6^A modification distribution and prevalence across the transcriptome of geese. Our results showed that the 3′UTR and CDS regions were the most highly modified regions, which is in agreement with other m^6^A reports in a recent review [2]. Notably, the P group exhibited higher m^6^A peak enrichment in 3′UTR region compared to the F and S groups. This finding is significant as it provides insight into the sites of RNA molecules that are most susceptible to m^6^A methylation. Knowledge of predominant locations, such as coding sequences, untranslated regions, or specific functional elements, provides indispensable context for understanding the functional implications of m^6^A modifications in geese.

In this study, compared to the P and F groups, the S group showed greater variability in the motif sequences. This heightened variability is likely attributable to its advanced developmental stage: livers in the S group had attained adulthood, whereas those in the P and F groups remained pre-adult. Consequently, this developmental disparity constitutes the primary biological basis for the observed differences—not only in motif sequences, but also in morphology and physiology. The dynamic regulation of RNA methylation across various growth phases in geese is a significant discovery, indicating that RNA m^6^A modifications are not static but rather responsive to the ever-changing requirements of developing tissues and organs. Knowing the mechanisms that control these changes is important for uncovering the molecular fundamentals of avian development.

Moreover, the study’s concentration on potential associations between m^6^A modifications and the developmental stages of geese is noteworthy. The results could shed light on how RNA methylation acts as a regulatory factor in the growth and maturation of these birds. For example, modifications in m^6^A levels in certain genes or transcripts may affect the timing or effectiveness of developmental processes, which could have implications for geese production and poultry biology research. In this study, using an integrative analysis of MeRIP-seq and RNA-seq, *CDK1* is identified as the potential core gene in the P group (birth stage). *CDK1* is a conserved gene in all organisms, plays vital roles in mitosis, and can drive the S phase without *CDK2* [24]. A previous study performed in mice showed that *CDK1* is invaluable for cell proliferation and embryonic development [25]. Hepatocytes with *CDK1* deficiency are incapable of mitotic entry. Nevertheless, following partial hepatectomy, *CDK1*-deficient livers undergo compensatory regeneration—mediated not by cellular proliferation, but by nuclear hypertrophy, as evidenced by a 28% increase in nuclear diameter and a concomitant 35% decrease in hepatocyte density [25]. Our multi-omics data suggest that m^6^A modification of *CDK1* may be associated with its expression and function during early liver development, although this speculative relationship requires direct experimental validation. In this research, the m^6^A-regulated cell cycle, p53 signaling pathway, and pyrimidine metabolism pathway were identified in liver tissue as novel potential targets for controlling geese growth and metabolism. Recently, *IGF2BP3* was found to play a role in regulating cell-cycle-related genes *CCND1* mRNA stability by directly interacting with its 3′UTR during hepatocarcinogenesis via m^6^A modification [26]. Another recent report found that *IGF2BP3* promotes lung tumorigenesis via attenuating p53 stability [3]. Moreover, knocking down *IGF2BP3* affected the expression of *CDK1* and the cell cycle process [27]. While these studies suggest a potential link between *IGF2BP3*, *CDK1*, and m^6^A, our data do not establish a direct causal relationship in goose liver development. Pyrimidine metabolism is a dynamic and versatile pathway involved in pathogens and cellular development [28]. Pyrimidine metabolism is how cells create and break down pyrimidine nucleotides, which are crucial components of DNA and RNA. It is worth mentioning that the cell cycle regulates the pyrimidine biosynthetic pathway, and the pathway is most active during the S phase of the cell cycle [29]. It is well known that both of these pathways (pyrimidine metabolism and p53 pathways) are involved in cell cycle regulation [29,30]; therefore, we speculate that the cell cycle pathway is the most crucial pathway involved in geese liver development. One research question that could be asked regarding goose development is which mechanism cell cycle pathway, p53 or pyrimidine metabolism, controls m^6^A in the developing liver at different growth stages.

From our top 10 to 15 KEGG pathway results, we noticed that the majority of the enriched pathways in the F and S periods belong to the metabolism of fats, immune-related pathways, and other substances. Indeed, the liver is a multi-functional organ that plays an essential role in lipid and cholesterol metabolism, immune function, glucose metabolism, nutrient storage, amino acid metabolism, detoxification, controlling growth signaling pathways through the endocrine system, and supporting diverse metabolic processes [15,16]. It plays a crucial role in lipid homeostasis, including de novo fatty acid synthesis, uptake of circulating lipids, triglyceride biosynthesis, and fatty acid oxidation for energy production [31]. A recent study in mice showed that when *Mettl3* is lost or removed, it leads to an increase in lipid accumulation [32]. In another study, knocking out *IGF2BP3* significantly reduced the accumulation of palmitic acid in keratinocytes of mice that had undergone m^6^A modification [33]. The liver serves as a critical immune organ, playing a central role in detecting and eliminating potential bloodborne pathogens while simultaneously maintaining a state of immune tolerance to prevent excessive immune responses [34]. Importantly, in hepatocellular carcinoma (HCC), the expression of lipid-metabolism-related genes was related to immune dysregulation [35]. Notably, *CDK1* is highly expressed in HCC tissues and exhibits a strong correlation with the infiltration of immunosuppressive cells, particularly regulatory T cells and myeloid-derived suppressor cells [36,37]. Furthermore, maternal obesity has been shown to inhibit hepatic lipid metabolism in offspring during weaning, with *CDK1* identified as a key regulator mediating this effect [38]. Collectively, our findings, along with previous research, suggest that the liver during the fast-growth stage may be primarily linked to the cell cycle (possibly through *CDK1*), while the liver during the fast-growth and sexual-maturation stages is more connected to fat metabolism and immune system regulation, all of which have undergone m^6^A modifications. However, these proposed connections are based on correlative multi-omics data and require functional validation. Subsequently, we will perform functional experiments at the cellular level by knocking down or overexpressing *CDK1* in primary goose liver cells. Changes in the expression of cell-cycle-related genes and in liver cell proliferation will be assessed to elucidate how m^6^A modification of *CDK1* regulates the development of goose liver cells.

## 5. Conclusions

This study provides insights into the intricate link between RNA m^6^A methylation and the developmental stages of male geese. It builds a platform for future research into the functional significance of m^6^A modifications in avian physiology and might have practical effects for improving the growth and health of poultry. In addition, this study contributes to the broader field of RNA epigenetics by extending our knowledge of m^6^A modification patterns. However, this study has limitations. The conclusions regarding the functional role of specific genes like *CDK1* and pathways are primarily based on bioinformatic predictions and multi-omics correlations, lacking direct experimental validation. Future work should include functional studies, such as in vitro or in vivo manipulation of candidate genes (e.g., *CDK1*, *IGF2BP3*) and m^6^A machinery, to establish causal relationships and elucidate the precise mechanisms by which m^6^A regulates liver development in geese.

## Figures and Tables

**Table 1:** Overview of MeRIP-seq and RNA-seq data.

Sample ID	Raw Reads	Raw Bases	Valid Reads	Valid Bases	Q20%	Q30%
P1_IP	39,504,498	5.54 G	39,503,018	5.54 G	96.37	90.72
P2_IP	31,737,610	4.45 G	31,736,460	4.45 G	96.43	90.91
P3_IP	33,336,602	4.68 G	33,335,440	4.68 G	96.58	91.15
F1_IP	37,997,214	5.34 G	37,995,918	5.34 G	96.43	90.89
F2_IP	36,448,374	5.12 G	36,447,258	5.12 G	96.41	90.86
F3_IP	35,763,900	5.02 G	35,762,682	5.02 G	96.49	90.99
S1_IP	36,112,440	5.08 G	36,111,454	5.08 G	96.50	91.00
S2_IP	35,641,788	5.01 G	35,640,382	5.01 G	96.39	90.77
S3_IP	36,782,326	5.17 G	36,781,014	5.17 G	96.52	90.98
P1_input	34,766,536	4.91 G	34,765,558	4.91 G	97.05	91.73
P2_input	38,232,604	5.40 G	38,231,848	5.40 G	97.10	91.85
P3_input	38,203,060	5.40 G	38,202,294	5.40 G	97.05	91.75
F1_input	38,735,304	5.45 G	38,734,404	5.45 G	97.07	91.84
F2_input	36,116,316	5.10 G	36,115,706	5.10 G	97.02	91.74
F3_input	38,454,342	5.43 G	38,453,444	5.43 G	97.05	91.77
S1_input	34,277,346	4.84 G	34,276,538	4.84 G	96.93	91.58
S2_input	34,772,422	4.91 G	34,771,522	4.91 G	97.04	91.72
S3_input	35,107,770	4.96 G	35,106,966	4.96 G	97.06	91.75

## Data Availability

All MeRIP-seq and RNA-seq data can be found in the Genome Sequence Archive (Genomics, Proteomics & Bioinformatics 2021) in the National Genomics Data Center (Nucleic Acids Res 2022), China National Center for Bioinformation/Beijing Institute of Genomics, Chinese Academy of Sciences (GSA: CRA012842), publicly accessible at https://ngdc.cncb.ac.cn/gsa, accessed on 23 September 2023.

## Supplementary Materials

The following supporting information can be downloaded at https://www.mdpi.com/article/10.3390/ani16060981/s1. Table S1. The percentage (%) of m^6^A peak distribution per gene. Table S2. KEGG enrichment analysis of DMGs in S vs. P. Table S3. KEGG enrichment analysis of DMGs in F vs. P. Table S4. KEGG enrichment analysis of DMGs in S vs. F. Table S5. S input vs. P input GO enrichment. Table S6. F input vs. P input GO enrichment. Table S7. S input vs. F input GO enrichment. Table S8. S input vs. P input KEGG enrichment. Table S9. F input vs. P input KEGG enrichment. Table S10. S input vs. F input KEGG enrichment. Table S11. The genes that exhibited significant differences in both methylation status and expression levels in S vs. P. Table S12. The genes that exhibited significant differences in both methylation status and expression levels in F vs. P. Table S13. The genes that exhibited significant differences in both methylation status and expression levels in S vs. F. Table S14. GO enrichment with the genes that exhibited significant differences in both methylation status and expression levels in S vs. P. Table S15. GO enrichment with the genes that exhibited significant differences in both methylation status and expression levels in F vs. P. Table S16. KEGG enrichment with the genes that exhibited significant differences in both methylation status and expression levels in S vs. P. Table S17. KEGG enrichment with the genes that exhibited significant differences in both methylation status and expression levels in F vs. P. Table S18. The primer information for qPCR validation.

## Abbreviations

m^6^A	N6-methyladenosine
MeRIP-seq	Methylated RNA immunoprecipitation sequencing
P	Post-hatch day 0
F	Fast-growth stage, 10 weeks old
S	Sexual-maturation stage, 30 weeks old
RNA-seq	RNA sequencing
PE150	Sequencing strategy, paired-end 150 bp
Q30	The percentage of bases with a quality score of 30 or higher
FPKM	Fragments per kilobase of exon model per million mapped fragments
DEGs	Differentially expressed genes
DMGs	Differentially methylated genes
GO	Gene Ontology
KEGG	Kyoto Encyclopedia of Genes and Genomes
UTR	Untranslated region
CDS	Coding sequencing
